# 10122 Development of an In Vitro in Vivo Correlation of Itraconazole Spray-Dried Dispersion Tablets

**DOI:** 10.1017/cts.2021.649

**Published:** 2021-03-31

**Authors:** Ana Luisa Coutinho, Asmita Adhikari, James Polli

**Affiliations:** University of Maryland Baltimore

## Abstract

ABSTRACT IMPACT: As the number of poorly water-soluble drugs in development increases, our research will expand on the science behind improving drug solubility and absorption and ensuring that promising poorly-water solubility drugs do not fail drug development. OBJECTIVES/GOALS: Spray-dried dispersion (SDD) tablet formulation is an approach to increase oral drug solubility and absorption. Methods to predict SDD performance in humans are poorly developed. We aim to develop an in vivo in vitro correlation (IVIVC) between in vitro dissolution and in vivo absorption of itraconazole SDD tablets. METHODS/STUDY POPULATION: This research project involves tablet manufacturing, in vitro dissolution experiments, and a clinical study. We manufactured fast-, medium-, and slow-release SDD tablets containing amorphous solid dispersion of itraconazole (100 mg) and different grades of the polymer hypromellose acetate succinate (HPMC-AS). Tablets differed in slug pressure, tablet compression force, and formulation composition. Dissolution studies were performed using the United States Pharmacopeia (USP) type II apparatus. The clinical study is an ongoing randomized, cross-over, open-label, fasted, single-dose trial in healthy participants (n=12). An IVIVC will be created by comparing the rank order of drug in vitro dissolution with in vivo absorption. RESULTS/ANTICIPATED RESULTS: Tablet manufacturing was successful, and the tablets displayed the same dissolution rate ranking order as anticipated. Fast-release tablets showed the highest percentage of drug dissolved by 10 min (74%) compared to medium- (62%) and slow-release (1.2%) tablets. Percentage drug dissolved differs by at least 10% at all time points among the different release-rate tablets. The clinical study is currently ongoing, and we expect that the pharmacokinetic (PK) profiles differ among the different tablets. We predict that the rank order of tablet absorption in humans will agree with the order of drug dissolved observed in the dissolution experiments. DISCUSSION/SIGNIFICANCE OF FINDINGS: Spray-dried dispersions are a formulation method to try to improve drug solubility and oral drug absorption. This research will elucidate manufacturing parameters that can impact tablet performance and expand on the ability of in vitro dissolution to predict human PK and streamline drug development of poorly soluble drug candidates.

